# P27 Investigation of subtherapeutic vancomycin levels

**DOI:** 10.1093/jacamr/dlae136.031

**Published:** 2024-08-23

**Authors:** Alice Liu

**Affiliations:** Gloucestershire Hospitals NHS Foundation Trust, UK

## Abstract

**Background:**

A retrospective audit was conducted in a large UK district hospital to assess the reasons behind subtherapeutic vancomycin assay levels and make recommendations as to how to improve the quality of vancomycin prescribing.

**Materials and methods:**

The vancomycin assay levels were extracted from biochemistry data retrospectively in a 6 month period and patients’ notes with prescription charts were examined for low assay levels as defined by the local hospital’s vancomycin protocol.

**Results:**

Twenty-seven percent of assay results (92 out of 337) were found to be at subtherapeutic levels in 57 patients, in which the vancomycin plasma concentration was less than 10 mg/L. Only 45 patient records were able to be retrieved for data analysis, of which 7 patients (16%) received either no or under loading dose during the treatment initiation. In terms of maintenance dose, 12 patients (27%) received under dose and 18 patients (40%) started the dose at the wrong time; half of this group had severely delayed the treatment for more than 4 h. Fifty-five subtherapeutic levels were identified among the 45 patients and the reasons for this included that assay samples were sent too early, dosage was prescribed incorrectly due to misconception of renal function, body weight miscalculation (i.e. ideal body weight versus actual body weight), as well as low dose being started because of acute or chronic renal impairment.

**Conclusions:**

The audit demonstrated there was a knowledge gap in vancomycin dosing calculation judged by patient’s body weight and renal function and the correct timing of assay. A pragmatic approach including education of junior doctors in antimicrobial prescribing, user-friendly guidelines or algorithmic chart in dosage recommendation, and pre-formatting of electronic prescriptions via EPMA should be adopted to increase the safe usage of vancomycin.

